# Canine cutaneous and subcutaneous soft tissue sarcoma in dogs: a consensus report from the Brazilian association of veterinary oncology

**DOI:** 10.3389/fvets.2026.1750148

**Published:** 2026-05-07

**Authors:** Carlos Eduardo Fonseca-Alves, Luiz Roberto Biondi, Denner Santos dos Anjos, Krishna Duro de Oliveira, Karen Batschinski, Sílvia Regina Ricci Lucas, Juliana Vieira Cirillo, Karine Germano, Ana Paula Luiz de Oliveira, Carmen Helena de Carvalho Vasconcellos, Heidge Fukumasu, Julia Maria Matera, Aline Machado De Zoppa, Priscila Emiko Kobayashi, Simone Carvalho dos Santos Cunha, Paulo Cesar Jark, Juliany Gomes Quitzan, Felipe Augusto Ruiz Sueiro, Marcelo Monte Mor Rangel, Rodrigo dos Santos Horta, Jorge Luiz Costa Castro, Simone Crestoni-Fernandes, Andre de Mattos Faro, Michelle do Carmo Pereira Rocha, Beatriz Furlan Paz, Adilson Paulo Marchioni Cabral, Fabrizio Grandi, Rafael Ricardo Huppes, Rodrigo Ubukata, Renée Laufer-Amorim, Maria Lucia Zaidan Dagli, Andrigo Barboza De Nardi

**Affiliations:** 1 Departamento de Cirurgia Veterinária, Faculdade de Medicina Veterinária e Zootecnia (FMVZ), Universidade de São Paulo-USP, São Paulo, Brazil; 2 Instituto de Oncologia Veterinária de São Paulo – IOVET, São Paulo, Brazil; 3 Faculdade Método de São Paulo-FAMESP, São Paulo, Brazil; 4 Universidade Metropolitana de Santos – UNIMES, São Paulo, Brazil; 5 VCLab Pathology, São Paulo, Brazil; 6 Safari Medicina Veterinária Especializada, São Paulo, Brazil; 7 Faculdade de Medicina Veterinária e Zootecnia, Universidade de São Paulo – USP, São Paulo, Brazil; 8 Hospital Veterinário Veros, São Paulo, Brazil; 9 E+ Especialidades Veterinárias, São Paulo, Brazil; 10 Departamento de Patologia, Reprodução e Saúde Única, Faculdade de Ciências Agrárias e Veterinárias, Universidade Estadual Paulista - UNESP, Campus de Jaboticabal, São Paulo, Brazil; 11 Hospital Veterinário Botafogo, Rio de Janeiro, Brazil; 12 Laboratório de Oncologia Comparada e Translacional, Universidade de São Paulo – USP, São Paulo, Brazil; 13 Universidade Anhembi Morumbi, São Paulo, Brazil; 14 Oncopet e Vet Radioterapia, Rio de Janeiro, Brazil; 15 Oncospes e Onconnectionvet Ribeirão Preto, São Paulo, Brazil; 16 Animalgoritmo, Campinas, Brazil; 17 Vet Câncer Care, São Paulo, Brazil; 18 Departamento de Clínica e Cirurgia Veterinárias, Escola de Veterinária, Universidade Federal de Minas Gerais – UFMG, Belo Horizonte, Brazil; 19 Pontifícia Universidade Católica do Paraná - PUCPR, Curitiba, Brazil; 20 Serviço de Cirurgia Veterinária, Instituto Federal Catarinense, Araquari, Brazil; 21 Departamento de Clínica e Cirurgia Veterinária, Faculdade de Ciências Agrárias e Veterinárias, Universidade Estadual Paulista - UNESP, Campus de Jaboticabal, São Paulo, Brazil; 22 Vetschool São Paulo e Virshow Patologia Digital, São Paulo, Brazil; 23 Centro de Oncologia e Especialidades Veterinárias - COEV, Maringá, Brazil; 24 Departamento de Clínica Veterinária, Faculdade de Medicina Veterinária e Zootecnia (FMVZ), Universidade Estadual Paulista -UNESP, Botucatu, Brazil; 25 Departamento de Patologia, Faculdade de Medicina Veterinária e Zootecnia, Universidade de São Paulo – USP, São Paulo, Brazil

**Keywords:** fibrosarcoma, guidelines, perivascular wall tumor, sarcomas, soft tissue

## Abstract

Soft tissue Sarcomas (STSs) are malignant mesenchymal neoplasms that affect the body’s connective tissues; they are common in dogs, especially in medium to large breeds and older animals. These tumors have poorly defined margins, making them invasive and prone to local recurrence. They metastasize at low rates and respond poorly to chemotherapy. STSs originate from the precursor of connective tissues, cartilage, and bones, and is characterized by spindle cells, specific histomorphological features, and immunohistochemical markers. Diagnosis and prognosis determination can be challenging due to the diversity of subtypes. International organizations, such as the Oncology Pathology Working Group, aim to standardize canine STS diagnosis and treatment. Therefore, it is necessary to standardize different procedures related to STSs for better comprehension of this tumor group in dogs. The Brazilian Association of Veterinary Oncology (ABROVET) organized a consensus event with Brazilian veterinary oncologists to discuss the main topics related to the STS approach. Thereafter, this document was organized as a literature review with consensus recommendations for clinical management, treatment, and future research.

## Introduction

The Brazilian Association of Veterinary Oncology (ABROVET) organized a Brazilian Consensus on canine soft tissue sarcomas (STSs), held in July 2023, to update and standardize the approach to these tumors. The event featured 25 speakers and 351 participants. On the first day, designated speakers critically reviewed each emerging topic. On the second day, seven experts from each field (diagnostic, prognostic, and therapeutic standards) reached a consensus opinion and defined guidelines for approaching canine STSs. All the attendees contributed to the development of the final consensus on the topic.

This paper presents the outcomes of the ABROVET consensus meeting. It is structured as a literature review that offers recommendations for handling clinical cases and future research.

STSs are a heterogeneous group of malignant mesenchymal neoplasms derived from “soft” connective tissue. They can occur anywhere in the body and most commonly affect the skin and subcutaneous tissues. STS constitutes 8–15% of cutaneous and subcutaneous tumors in dogs, and is prevalent in middle-aged to elderly dogs and medium or large breeds (1–4). STSs are classified as sarcomas because of their mesenchymal origin. The literature has shown a good prognosis for this tumor group, and the term “sarcoma” has been under debate (5, 6). For this reason, a key question debated among specialists was whether they should be classified as soft-tissue sarcomas or soft-tissue tumors. Their histologic diagnosis is often ambiguous and requires careful morphological examination, complemented by immunohistochemistry. After a two-day discussion at the ABROVET event, we concluded that this tumor group should be called STS or Soft Tissue Tumors (STTs) when included benign counterpart (5, 6).

In this consensus, we adopted the terminology STS and STT following the most recent classification proposed for tumors of domestic animals (7). In this framework, the term STT is used as a broad designation encompassing all lesions arising from soft tissues, including non-neoplastic proliferations and both benign and malignant neoplasms. The term soft tissue neoplasm (STN) refers specifically to neoplastic lesions of soft tissues, regardless of biological behavior, whereas STS is reserved exclusively for malignant mesenchymal tumors.

This nomenclature allows a clearer distinction between benign and malignant entities and aligns with contemporary veterinary surgical pathology classifications. In clinical practice, most soft tissue neoplasms diagnosed in dogs correspond to low-grade sarcomas (grade I or II), which typically exhibit locally invasive behavior with relatively low metastatic potential, whereas high-grade tumors (grade III) represent a smaller proportion of cases but are associated with increased metastatic risk and more aggressive biological behavior (5, 6).

## Classification of soft tissue tumor subtypes

STTs are neoplasms of mesenchymal origin; that is, they are part of a diverse group of neoplasms that can originate from muscle, neurovascular, connective, and adipose tissues (6–8). Among the histological subtypes that make up this group of tumors, fibrosarcomas, malignant nerve sheath tumor, perivascular wall tumor, myxosarcomas, liposarcomas, and undifferentiated sarcomas are most frequently described (9).

### Fibrosarcomas

Fibrosarcoma is one of the most prevalent STSs in dogs, with an 18 to 23% prevalence among the STSs (8, 10). Some breeds, such as the Doberman, Rottweiler, and Setter, are more susceptible to fibrosarcoma (8, 11). Fibrosarcomas can occur spontaneously or may develop secondary to injections or microchip implantations. When they occur secondarily, they are often in the subcutaneous tissue, with peritumoral infiltration of lymphocytes (12). When they are spontaneous, they can affect the limbs, trunk, abdomen, face, and eyelids (13, 14).

Fibrosarcomas present with well-or poorly differentiated histological characteristics, pleomorphic spindle cells, and mitotic figures. The cells organize themselves into intertwined bundles that form a herringbone pattern on a background of dense collagenous stroma (4, 12). Immunohistochemically, fibrosarcomas differ from fibromas by stronger expression of Ki67 and endosialin, and the absence of COX2 in immunostaining (15). The expression of Ki67 and endosialin is positively correlated with the histological grade, and the presence of endosialin may favor the metastatic potential of a tumor by increasing its proliferative capacity, as well as the presence of Ki67 (16). Furthermore, fibrosarcoma express platelet-derived growth factor alpha (PDGF-A) with cytoplasmic and nuclear staining for its receptor, along with positive staining for the integrin alpha V subunit. The expression of PDGF-A and its integrin alpha V subunits was positively correlated; this contributes to tumor progression (12).

No HRAS, KRAS2, or NRAS mutations were found in canine fibrosarcoma samples (17).

A recent proteomic study of canine fibrosarcoma neoplastic and peritumoral tissues identified 25 proteins expressed solely in the tumor tissue in more than 80% of the samples analyzed (14). This information could be useful for the evaluation of future targeted therapies for fibrosarcoma in dogs, as it demonstrates the presence of proteins expressed in other STSs, which already have pilot studies testing target drugs in humans (18, 19). Supplementary Table 1 summarizes key information about these genes.

### Myxosarcomas

These are tumors of fibroblastic origin with a significant myxoid matrix formed by mucopolysaccharides, featuring stellate or spindle-shaped cells in the mucinous stroma (4, 20, 21). The cutaneous form affects the head, trunk, and limbs. Young animals can be affected by myxosarcoma, with cases reported in dogs aged 3.5–14 years, with a median age and weight of 10 years and 25 kg, respectively (22). A retrospective study of 32 dogs with myxosarcoma reported a median survival of 730 days (20–2,345 days), regardless of the type of treatment adopted. Nonetheless, for animals with mitotic counts exceeding 10 mitotic figures per field, the survival time was reduced to 433 days. In contrast, when the mitotic number was lower than 10, the median survival time was 1,393 days. In 40% of the animals, there was local recurrence after tumor resection, with a median recurrence period of 115 days; 25% showed metastases to lymph nodes or lungs (22).

## Peripheral nerve sheath tumors

Peripheral nerve sheath tumors (PNST) occur in various soft tissues and organs that have peripheral innervation. They are histologically formed by Schwann cells, perineural cells, and intraneural fibroblasts, whose pleomorphic neoplastic cells are fusiform or polygonal and organized in short, intertwined fascicles in a storiform pattern, with nuclear palisades or sheets and collagenous stroma (23).

PNST are classified by their morphological and malignant characteristics, and are differentiated into benign and malignant tumors. They include histological types such as schwannomas, neurofibromas, perineuriomas, traumatic neuromas, and malignant tumors of the peripheral nerve sheath (24, 25).

Benign tumors may present a histological pattern similar to the classic model of human schwannomas or neurofibromas. They tend to be well-delimited and located in the skin and subcutaneous tissue, with an equal distribution of Antoni A and B histological patterns (23, 25). The Antoni A histological pattern is composed of compact spindle cells organized in bundles with storiform and palisade patterns. In the Antoni B area, spindle cells are loosely arranged within a myxoid background (26). Malignant subtypes are often subcutaneous, poorly delimited, invasive in deeper tissues, show necrosis, and are associated with high rates of local recurrence and relatively low survival times (23, 25).

A Brazilian study investigated 2,984 naturally occurring skin neoplasms in dogs and PNST constituted 2.34% of all these tumors. Considering only dogs with STTs, PNST were observed in 69% of cases, in 21% of recurrence cases, and in 11% of dogs with metastases. Among the PNST cases, a higher prevalence was observed in mixed-breed dogs (43%), followed by German Shepherds (10%) (25). A study to identify possible target drugs for PNST observed that all samples (*N =* 10) of malignant neoplasms expressed receptors for platelet-derived growth factor *β* (PDGFR- β), with 80% expressed in more than 25% of tumor cells. Of the benign tumors evaluated (*N =* 9), only three samples expressed this receptor in more than 25% of the cells (33.3%). The Ki-67 index was not significantly different between benign or malignant tumor subtypes, and was not related to PDGFR-*β* expression levels. However, tumor size was associated with malignant neoplasms, and the study findings suggest that the therapeutic use of tyrosine kinase inhibitors may be beneficial, given the significant expression of the PDGFR-β receptor (27).

### Perivascular wall tumors

Perivascular wall tumors (PWTs) are a subgroup of the STSs, with specific histological and immunophenotypic patterns that tend to exhibit less aggressive clinical behavior compared to that of other STSs, representing 6.3% of an STS series in dogs (8, 28). Perivascular tumors are neoplasms arising from the mural cells of blood vessels, excluding the endothelial lining, and include glomus tumors and perivascular wall tumors (PWTs), which comprise hemangiopericytoma, myopericytomas, angioleiomyomas, angiomyofibroblastomas, and angiofibromas (4, 29).

PNST have been controversial in veterinary tumor classification due to their similarity to human oncology features, as they are composed of subcutaneous spindle cells characterized by spiral growth patterns. However, some authors sought to differentiate perivascular wall tumors from PNST because their cellular, perivascular, or perineural origins have been questioned, in addition to the morphological difficulties often encountered in this differentiation (30).

A Brazilian study sought to differentiate 71 STT cases with morphological characteristics compatible with PWTs and PNST, indicating that the PWT morphological pattern was not sufficiently indicative for the differential diagnosis of these tumors, with 16.9% of the samples being positive for neural and muscular markers (31). Consequently, several studies have sought to identify immunohistochemical markers that differentiate STTs and better classify and type perivascular wall tumors (31–33).

The morphological characteristics of PWTs include vascular growth patterns such as staghorn, placentoid, pericapillary whorl, tunica media, adventitious whorl, and tunica media radiating bundles. The presence of intertwined or parallel solid storiform myxoid bundles that mimic Verocay cells (nuclei organized in parallel rows in a palisade) has also been observed; additionally, giant cells, necrosis, hemorrhages, and multitobulations may be present (31). PWTs affect dogs with a median age of 10 years (3–17 years) and a weight of 28 kg (3.5–52), with the most represented breeds being Mixed Breed dogs (30%), followed by Boxers (15%), and German Shepherds (9.4%) (34).

### Liposarcomas

Liposarcomas are locally invasive STSs that rarely metastasize, occurring in dogs at a frequency of 0.1% among all tumor types and 1.7% among STSs (8, 35, 36). They can be subdivided into well-differentiated, pleomorphic, myxoid and undifferentiated (37). They develop from adipose tissue, in which lipoblasts or lipocytes are organized as polygonal cells with a vacuolated cytoplasm (4).

Different subtypes of liposarcoma express proto-oncogenes in varying ways. MDM2 was expressed in samples from well-differentiated (100%) and undifferentiated tumors (75 to 100%), whereas only one sample from myxoid neoplasia (*N =* 7) and none from the pleomorphic subtype were positive for MDM2 (37, 38). Knowledge of MDM2 immunoexpression allows for the reclassification of well-differentiated samples as pleomorphic liposarcomas (38, 39). Myxoid tumors are associated with p53 expression, which correlates with higher Ki67 expression and mitotic counts. There was low expression of p53 in liposarcomas, as only 11.6% of samples were positive, with none being positive in the undifferentiated and pleomorphic subtypes (37).

Liposarcomas can also affect young animals aged 2 to 16 years, with masses in the axial skeleton (46%), appendicular (41%), or viscera (10.7%), and an average size of 6.8 cm (0.5–20 cm) (35). When evaluating liposarcomas using tomographic imaging, focal areas of fat attenuation are not mandatory for characterizing the tumor mass, as they were absent in 23% of the samples evaluated. On computed tomography (CT), liposarcomas are distinguishable from infiltrative lipomas by enhancement with contrast medium (40–42). They may present with heterogeneous internal attenuation, absence of a peritumoral capsule, and mineralization foci (41, 42). Liposarcomas showed high expression of fibroblast growth factor type 2, fibroblast growth factor receptor 1, and PDGF-*β* (81.6%); few cases expressed c-kit (39).

### Malignant mesenchymomas

Malignant mesenchymal tumors can arise from any mesenchymal tissue and are composed of more than one cell type and multiple extracellular matrix components, including osteoids, chondroids, and collagen (4). In tumor morphology, adipose tissue, osteoblastic cells, necrosis, and cells with pleomorphic nuclei can be identified (43, 44). Due to the multiplicity of tissue components, incisional biopsy samples may be inaccurate for correct diagnosis of this type of STS (44, 45).

In general, diagnosis can only be provided postmortem due to limitations in histopathological examination or euthanasia of severely affected animals (44, 45). Malignant mesenchymoma can exhibit aggressive behavior, with metastatic dissemination into the bone marrow (44).

### Undifferentiated pleomorphic sarcomas

Undifferentiated pleomorphic sarcomas (UPSs), previously known as malignant fibrous histiocytomas, originate from fibrous tissue and primitive mesenchymal cells that have the potential to develop into fibroblasts or myofibroblasts (4, 46). This category comprises multinucleated fibroblastic, karyomegalic, cytomegalic, or histiocytoid cells organized in a storiform pattern with variable inflammatory infiltrate (4).

UPS can be divided into the following subtypes: inflammatory, with occasional bizarre histiocytoid cells and a significant inflammatory infiltrate composed of lymphocytes, plasma cells, eosinophils, and neutrophils; giant cell, with numerous multinucleated giant cells next to spindle and mononuclear histiocytic cells; and storiform UPS-pleomorphic cells, with cells similar to fibroblasts in a storiform arrangement and karyomegalic or multinucleated histoid cells, with an inflammatory infiltrate around them (47).

Some retriever breeds were considered predisposed to UPS; however, the predisposition of retriever breeds is not consensual. In the analysis of 110 STSs of unclassified Golden Retrievers, only one case related to UPS was observed (0.9%), whereas another study identified the tumor in 100% of retriever breeds (*N =* 14) (47, 48).

Genomic studies have demonstrated that aberrant DNA methylation patterns characterize the UPSs. However, cell culture analyses of hypomethylating agents showed ineffective treatment (16). Immunohistochemical analysis of vimentin and major histocompatibility complex class II was positive in UPS samples to classify unidentified STSs, whereas there was no expression of cytokeratin, Myo D1, S100, or von Willebrand. Desmin and actin expression was variable in non-neoplastic tissues in the sample (47).

### Leiomyosarcomas

Leiomyosarcomas originate from smooth muscle and are composed of leiomyoblasts, leiomyocytes, and cells with “cigar-shaped” nuclei and prominent cytoplasm (4). Histologically, they can mimic other mesenchymal neoplasms, and distinctions between leiomyoma and leiomyosarcoma are based on infiltrative growth, necrotic areas and mitotic count. However, in veterinary medicine there is no cut off for these histopathological features (49, 50). In humans, tumor grading guidelines are available based on cellularity, nuclear atypia, mitotic count and percent necrosis, but other factors such as location, neurovascular invasion and anatomic compartment of origin are prognostic factors (51).

Although no definitive gold standard has been established for the diagnosis of canine leiomyosarcoma, immunohistochemical staining is often required to confirm the diagnosis and to exclude other more frequently encountered tumor types (50). As many other mesenchymal neoplasms, leiomyosarcoma is positive to vimentin (50, 52–54). Other expressions of cellular markers are used to aid determining the leiomyosarcoma phenotype, including SMA (smooth muscle actin), desmin and calponin (4, 55, 56). SMA is an actin isoform, normally restricted to smooth muscle cells, myofibroblasts and myoepithelial cells (57). Desmin is an intermediate filament protein of endothelial and all muscle cells types, including smooth muscle cells, skeletal cells and cardiomyocytes (58). Negative immunostaining for SMA and desmin does not exclusively exclude a diagnosis of leiomyosarcoma (56). Calponin is cytoskeleton-associated actin-binding proteins restricted to smooth muscle marker and myoepithelial cells (56, 59, 60). However, the application in veterinary diagnostic remains limited (56).

## Incidence, risk factors and epidemiology

Global studies indicate a predominance of subcutaneous or musculoskeletal locations of STTs, compared to other sites. Additionally, they may represent approximately 15% of all cutaneous and subcutaneous tumors in dogs (61–63). Middle-aged to senior dogs (7–15 years) are most affected, as are medium-to-large-sized dogs. However, no breed or sex predispositions were identified (62).

The incidence of STSs in dogs is 35 per 100,000 animals and risk factors associated with STSs include radiation, trauma, orthopedic implants, and foreign bodies (62). STSs have been linked to parasitic infections, such as the presence of *Spirocerca lupi* (64). However, the cause of STS oncogenesis in dogs remains unclear (1, 3). Most STS lesions are in cutaneous or subcutaneous tissues, exhibit locally invasive behavior, and tend to infiltrate the fascial planes (8, 65). Up to 60% of canine STSs occur in the limbs; other anatomical locations are less affected, including the trunk (35%) and head or neck (5%) (63).

STSs are generally characterized by a high rate of local recurrence in high-grade tumors, and a low rate of metastasis. An important characteristic of these tumors is that metastasis occurs via the hematogenous route, affecting approximately 20% of patients (64, 65).

A Brazilian epidemiological study of 30 dogs found 80% of the STSs were in the subcutaneous connective tissues (66). However, no large-scale study has been conducted in Brazil assessing breed predisposition in dogs with STSs. Females were more frequently affected than males (*N =* 68 vs. *N =* 37), and an association was found between sex and tumor location (66). Females had a higher frequency of tumors on the trunk, and males had a higher frequency of tumors on the limbs. This may be related to the expression of estrogen and progesterone receptors in different cells, with females being more predisposed. The expression of estrogen receptors (ERs) and progesterone receptors (PRs) was previously evaluated in samples of PNST and perivascular wall tumors (PWTs), identifying PR expression in 80 samples (100%); none of the tumors expressed ER. The expression of PRs was positively correlated with the STS grade and cell proliferation index (Ki67), which may be related to tumor growth (31). In women with STSs, ER expression is a positive indicator of survival, whereas PR expression is indicative of lower survival rates in affected men (67).

*Consensus recommendation*: There is still insufficient evidence to confirm the predisposition related to the role of different reproductive hormones, and new studies are needed to confirm this hypothesis.

## Genetic factors

The new classification of human STSs, by the World Health Organization (WHO) in 2020, was based on the molecular characteristics of neoplastic tissues to improve the diagnosis of poorly differentiated tumors with similar morphological characteristics, considering that more than 100 histological subtypes have been described (68, 69). Similarly, the genomic and transcriptomic profiles of canine STSs can aid in the differentiation of the mesenchymal neoplasms that constitute this group (70).

Studies have been conducted to evaluate specific histological subtypes of canine STSs, rather than studies of STSs as a group of tumors. Among the histological subtypes with the greatest number of genomic and transcriptomic studies are fibrosarcoma (12, 15, 71), PWTs (15, 70), perineural sheath tumors (15, 70), leiomyosarcomas (72), and undifferentiated pleomorphic sarcomas (UPSs) (16). Evaluating genetic alterations that may lead to tumorigenesis and progression of STSs helps identify useful markers for histologic differentiation of different tumor subtypes. Thus, they allow clinicians to predict the behavior of the disease and establish the necessary treatment, promoting a better quality of life and survival for patients (71). As with human STSs, molecular studies must be conducted homogeneously between the different histological subtypes to confirm that each histologic subtype is molecularly similar, to enable grouping as molecularly similar entities.

Due to recent advances in tumorigenesis research, more than 3,000 genes have been identified in different samples of canine STSs, including 2,090 genes specific to fibrosarcoma, 875 specific to PWTs, and 291 specific to PNST. In the analysis of the three tumors listed above, greater genetic similarity was identified between tumors of the perivascular wall and peripheral nerve sheath, distinguishing them from fibrosarcomas. Fibrosarcomas show higher expression of transcription factors such as GDF11, whereas PNST show fewer genes related to mitosis (71).

Some genes exhibited higher expression levels in PNST, such as FMN2, KIF1B, GLI1, ROBO1, NMUR2, DOK4, and HMG20B, which are involved in neuroectodermal differentiation. Genes associated with carcinogenesis include FHL2, PLAGL1, FNBP1L, BAG2, HK1, CSK, and Cox5A (73).

*Consensus recommendation*: from a molecular perspective, this consensus indicates that further genomic and transcriptomic studies, of each histological subtype within canine STTs, are necessary to group the subtypes. Thus, molecular subdivisions of these tumors could be established within the STT group.

## Clinical signs

Symptoms vary according to the size and invasiveness of the lesion and its appearance (whether ulcerative or not), depending on the affected anatomical structure (4). In general, canine STTs are painless; however, when they present with an ulcerative appearance, they cause pain and discomfort (61). Abdominal STTs can compress organs such as the rectum, stomach, urinary bladder, and urethra, which can cause tenesmus, vomiting, slow gastric emptying, urinary retention, or pollakiuria, depending on the affected organ. In cases with splenic and liver involvement, tumor lysis can cause peritoneal hemorrhages in addition to liver dysfunction (65). Some patients may experience pain and difficulty when moving around if the mass is located in joint regions. Neoplasms in the cervical or mediastinal regions can cause inspiratory dyspnea, dysphagia, and regurgitation due to obstruction caused by compression of the airways and esophagus (8).

## Diagnosis

### Cytological diagnosis

The differential diagnosis of STTs can be challenging because of the considerable morphological overlap between different types of STTs. Furthermore, cytological differentiation between STTs and reactive lesions, such as granulation tissue, focal reactive hyperplasia, inflammatory myofibroblastic tumor, and fasciitis, is difficult. However, cytology continues to be used as an initial method for screening tumor lesions (8).

Cytologically, STT cells vary from spindle-shaped to dendritic (stellate), epithelioid, and even rounded. Typically, they are exfoliated individually or in storiform groups. Given that mesenchymal cells also predominate in areas of inflammation, accurate interpretation of cytology requires a correlation with the clinical appearance and location of the mass. A study evaluated cytology aspiration in 40 dogs with STSs, and revealed inconclusive or inconsistent diagnoses in 38% of the cases (74). Another study demonstrated a correlation between cytological classification scores (averaged across observers). Twenty-one patients were included in the study (10 with Grade I STSs, nine with Grade II, and two with Grade III). The number of mitotic figures (≥3) per 200 cells was the only parameter that showed a significant but weak positive correlation with histological grade. No Grade I tumors had ≥3 mitotic figures per 200 cells; ≥3 mitotic figures per 200 cells were observed in 33% of Grade II tumors and 50% of Grade III tumors (75). This study suggested that an increase in the number of mitotic figures observed on cytology may be correlated with Grade II and III STSs; however, the sensitivity of this parameter for classifying STS grades appear to be low (76).

Another study evaluated the utility and accuracy of a cytology grading system for fine-needle aspiration cytology smears of cutaneous and subcutaneous STSs in 33 canine and feline cases (77). Canine STSs showed cytohistological agreement in 12/20 cases (60%). Agreement was observed in 4/8 (50%) Grade 1, 8/12 (67%) Grade 2, and 0 cases of Grade 3. Agreement was observed in 5/6 (83%) Grade 1, 4/4 (100%) Grade 2, and 2/3 (66.6%) Grade 3 cases. This agreement is very similar to results in human medicine (77).

*Consensus recommendation*: the authors of this consensus indicated that there are major limitations to cytological examination and that the definitive diagnosis of STT must always be made through histopathological analysis. However, it is important to highlight that, owing to morphological overlap, we are often unable to define STTs only on histopathology (76, 77).

### Histopathological diagnosis

For a definitive diagnosis, incisional biopsy is the method of choice in STT cases, before definitive treatment. An important point of discussion is that, in histopathological analysis, areas of necrosis or necrotic regions are avoided when collecting biopsy samples. However, necrosis is assessed and considered important for grading STTs. Regarding the performance of biopsies, between 12 and 29% of cases in which the diagnosis is made through preoperative biopsies may exhibit an erroneous histopathological grading when compared with the results of total analysis of the tumor after complete excision (75).

*Consensus recommendation*: this consensus recommends biopsy before the excision of large masses or tumors with unresectable surgical margins for histopathological evaluation and surgical planning. However, after complete tumor removal, the sample must be sent for analysis again to obtain more reliable grading since tissue biopsies provide small tissue fragments.

The histopathological STT grading is based on three distinct criteria: the cellular differentiation score, mitotic count (MC) score, and tumor necrosis score (Table 1) (4). It is worth noting that the mitotic count must occur in 10 fields at 400 × magnification, totaling an area of 2.37 mm^2^. The count must be performed at a hotspot and continue in 10 continuous fields. A score was assigned to each of the criteria to grade the injury, and points were added. The histological grade is defined as the sum of individual scores (78).

**Table 1:** Histological grading system for cutaneous and subcutaneous soft tissue sarcomas.

Differentiation score
Score 1: Well-defined diagnostic pattern for classification (well-differentiated) Score 2: Poorly defined diagnostic pattern, but sufficient for classification (poorly-differentiated) Score 3: Undifferentiated sarcoma, sarcomas of unknown type
Mitotic count
Score 1: 0–9 mitoses per 10 HPF* Score 2: 10–19 mitoses per 10 HPF Score 3: >20 mitoses per 10 HPF
Tumor necrosis – assessed in histologic section(s)
Score 0: No necrosis Score 1: ≤ 50% tumor necrosis Score 2: > 50% tumor necrosis
Histologic grade: sum of the scores
Low Grade: Total score 2, 3 Intermediate Grade: Total score 4, 5 High Grade: Total score >6

*10 high-power fields (HPF: 40X objective and 10X ocular with FN 22) measures 2.37 mm^2^ with a microscope. Select an area at the periphery with the most active cellular. Source: Dennis et al. (4).

However, the classification of STT grades may have interobserver divergence because the percentage of necrosis and the degree of tumor differentiation are highly subjective (79) and dependent on the macroscopic sampling performed (which may have avoided areas of necrosis). High interobserver variability has been reported in the classification of necrosis, which may result in complications for treatment plans (80). Therefore, artificial intelligence algorithms, particularly machine learning, have emerged to improve classification by automatically detecting regions of necrosis (79). In the automatic identification of tumor necrosis on digitized slides of canine soft tissue tumors, the diagnostic accuracy was 92.7%. This is a promising tool to minimize human error in the assessment of necrosis in STTs, and increase the efficiency and accuracy of histopathological classification (81).

Grade I STT is the most common form of STT in many regions and is generally a slow-growing lesion with the lowest recurrence rate after surgical excision. Grade II tumors, although associated with a slightly higher risk of local recurrence, are often considered clinically similar to Grade I tumors due to their relatively indolent behavior, low metastatic potential, and curability with adequate local tumor control (66). In contrast, Grade III STTs are biologically more aggressive, associated with higher rates of metastasis, significant morbidity, and, in some cases, life-threatening outcomes. Therefore, they require a distinct clinical approach (82).

*Consensus recommendation*: this consensus recommends that histopathological grading of soft tissue mesenchymal tumors should strictly follow the established three-tier system based on cellular differentiation, mitotic count, and tumor necrosis. The MC must be assessed in 2.37 mm^2^ total area, starting from the most mitotically active region. To minimize interobserver variability, it is strongly advised that the evaluation of necrosis and differentiation be performed by experienced pathologist, ideally supported by standardized sampling protocols to include necrotic regions when present.

### Immunohistochemistry

Diagnostic immunohistochemistry (IHC) is commonly used to evaluate STTs. It is best used as a diagnostic adjunct after careful evaluation of the histopathology and differential diagnosis. The effective use of immunohistochemistry specifically evaluates the differential diagnostic possibilities. In human medicine, a small “universal” panel of CD34, desmin, epithelial membrane antigen, keratin cocktail AE1/AE3, protein S100, and smooth muscle alpha actin is recommended (83). These markers assist in the differential diagnosis of fibroblasts, myoids, nerve sheaths, perineural cell tumors, and epithelioid sarcomas. However, they are all multi-specific; therefore, it is necessary to identify their distribution in normal and neoplastic tissues.

At the research level, it is essential to conduct new, well-designed prospective studies that include immunohistochemical analyses to differentiate histological subtypes, along with independent pathological review to confirm whether STTs constitute a homogeneous group of tumors. The consensus recommends immunohistochemistry to characterize the cellular origin specifically for research purposes. In current clinical practice, however, there is no evidence that immunohistochemical classification alters prognosis or therapeutic decision-making for patients diagnosed with STTs. Additionally, it is critical that future studies clearly distinguish between all-cause mortality and STT-related mortality, as this distinction directly impacts the interpretation of survival analyses. The use of survival as a primary outcome measure in tumors that rarely result in death or euthanasia must be approached with caution. Censoring in survival curves should only apply to patients who died from causes unrelated to the tumor, while disease-specific mortality should be used to provide a more accurate assessment of prognosis and treatment efficacy. These considerations highlight the urgent need for prospective, standardized data to improve clinical relevance and comparability between studies.

*Consensus recommendation*: This consensus does not recommend the routine use of immunohistochemistry for the classification of canine soft tissue sarcomas. Histopathological evaluation remains the gold standard for diagnosis and grading, and IHC should be reserved for selected cases in which specific differential diagnoses need to be investigated. IHC should be used primarily as a research tool or as an adjunct method in selected cases after thorough histopathological evaluation and consideration of differential diagnoses. Currently, there is insufficient evidence to support the routine use of immunohistochemistry for the diagnostic classification of canine soft tissue tumors in clinical practice.

## Staging

Regarding STT staging, it follows the standard for solid neoplasms, with a system for evaluating tumor size (T), regional nodal involvement (N), and distant metastasis (M) (84). A study proposing a unified staging system for STTs in dogs evaluated 105 dogs with STTs that were treated with surgery alone. They evaluated samples from patients with lymph node metastases and found a significant association between regional lymph node metastasis and overall survival (OS). Patients with lymph node metastases did not survive for more than 120 days. The staging systems proposed by the authors are presented in Tables 2, 3. They found an association between disease stage and animal survival. Dogs affected by STT Stages I and II have greater survival than those at Stages III and IV (66).

**Table 2:** TNM system for canine cutaneous and subcutaneous soft tissue sarcomas, adapted from the human classification retrieved from previous literature (52).

Definition of primary tumor (T)	
T category	T criteria
Tx	Primary tumor cannot be assessed
T0	No evidence of primary tumor
T1	Tumor 3 cm or less in greatest dimension
T2	Tumor more than 3 cm and ≤7 cm in greatest dimension
T3	Tumor more than 7 cm and ≤12 cm in greatest dimension
T4	Tumor more than 13 cm in greatest dimension

Definition of regional lymph node (N)	
N category	N criteria
N0	No regional lymph node metastasis
N1	Regional lymph node metastasis

Definition of distant metastasis (M)	
M category	M criteria
M0	No distant metastasis
M1	Distant metastasis

Definition of Grade	
Gx	Grade cannot be assessed
G1	Total differentiation, with tissue resembling the normal counterpart, mitotic count ≤9, no necrosis
G2	Total differentiation, with tissue showing poor differentiation, mitotic between 10 and 19, necrosis ≤ 50%
G3	Undifferentiated sarcoma, mitotic count ≥ 20, necrosis ≥50%

**Table 3:** New clinical staging system for dogs affected by cutaneous and subcutaneous soft tissue sarcomas, retrieved from previous literature (52).

Stage	T	*N*	M	Histological grade
Stage I	Tx, T1 or T2	N0	M0	Gx or G1
Stage II	T2, T3 or T4	N0	M0	G2
Stage III	T3 or T4	N0	M0	G3
Stage IV	Any T	N1	M0	Any G
	Any T	N0	M1	Any G

The staging system can be complex to apply as it must consider the methods for defining nodal involvement and distant metastasis.

Defining the ideal test for evaluating distant metastasis is crucial. Advanced imaging techniques like computed tomography and magnetic resonance imaging help with surgical planning, as they allow for better assessment of tumor size and infiltration (85, 86).

Computed tomography and magnetic resonance imaging are also fundamentally important for diagnosing PNST involving the plexuses (brachial and lumbosacral) and medullary roots. Magnetic resonance imaging is superior to computed tomography for detecting plexus tumors due to its excellent soft tissue contrast resolution (87).

*Consensus recommendation*: based on the available literature, the consensus recommendation applying the proposed staging system (66) and suggests further studies to confirm its clinical applicability.

## Local therapeutic approach

Understanding the development and biological behavior of STTs in dogs leads to more radical and effective local control therapies and adjuvant systemic therapies can promote longer-lasting control of disease progression (62).

### Surgery

Surgical excision remains the gold-standard treatment for STTs, with the objective of removing the tumor together with a margin of grossly normal tissue to allow histological confirmation of complete resection. Inadequate surgical removal is directly associated with more rapid disease recurrence (4). Several studies consider obtaining adequate margins in the first surgical intervention is an important prognostic factor for STTs (4, 8, 21, 61). The surgical margin will be determined based on the patient’s size and history, presence of comorbidities, and behavior. Reinforcing the importance of biopsy for histopathological evaluation, grading and surgical planning.

*Consensus recommendation*: after reviewing the literature, this consensus document defines the recommended wide surgical margins for STT as follows: Grade I tumor smaller than 3 cm should be excised with 2 cm lateral margins and one deep fascial plane; Grade II tumors require 3 cm lateral margins with one deep fascial plane; and Grade III tumors, excision should include 5 cm lateral margins are 5 cm and two deep fascial planes (62, 88).

**Figure 1:**
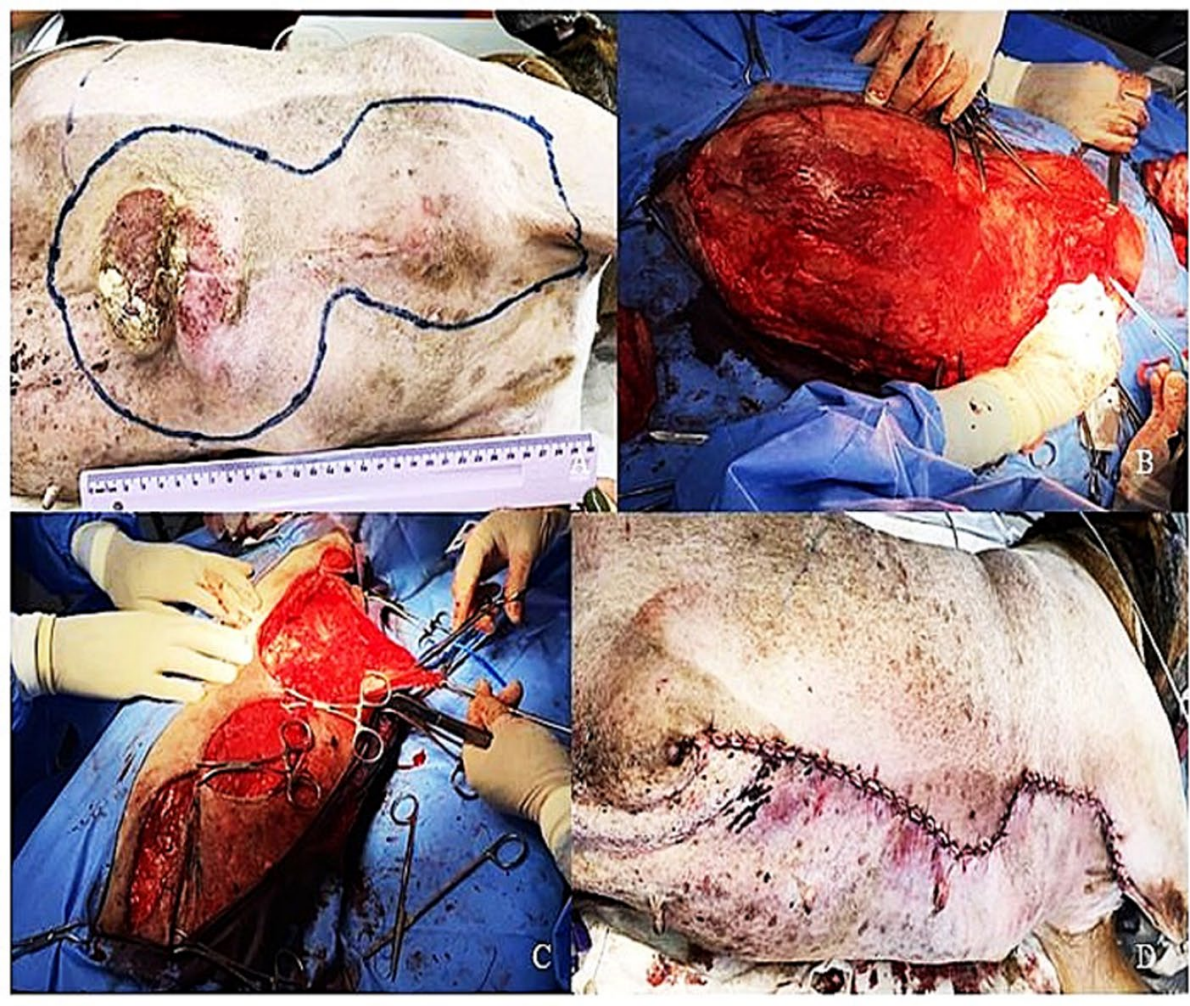
Reconstructive surgery of a canine soft tissue tumor. **(A)** Ulcerated lesion in the abdomen, with diagnosis of soft tissue tumor, grade II. **(B)** Surgical bed after en bloc resection of the mass. **(C)** Reconstructive surgical closure by first intention using different techniques. **(D)** Final appearance of the sutures after complete tumor removal.

Margins between 2 and 3 cm were effective for most STSs (89). Some Grade III tumors may require a more aggressive approach because of their highly recurrent behavior, to avoid incomplete resection. For the procedure to be successful, a good surgical technique needs to be followed, using associated reconstructive techniques (Figure 1), adequate surgical antisepsis, pain control, and satisfactory postoperative care, as surgical approaches for STSs tend to be extensive (90, 91).

Potential confounders, such as tumor size, grade, and disease burden at the time of surgery, may influence outcomes. Furthermore, many studies do not clearly distinguish between all-cause mortality and tumor-related mortality, which can significantly impact the interpretation of survival data. While there is some concern that extensive and invasive procedures could facilitate hematogenous dissemination of neoplastic cells, this hypothesis remains controversial and insufficiently supported by current evidence (21, 90, 92). Given that the majority of soft tissue mesenchymal tumors are classified as low-grade (Grade 1 or 2), conservative surgical excision is often sufficient to achieve local control and may be curative in many cases, especially when adequate margins are obtained. This reinforces the importance of individualized surgical planning based on tumor grade and biological behavior.

### En bloc resection

En bloc resection constitutes the main therapy for local control, with successful excision reducing the chance of recurrence, and increasing survival times. It involves measuring the desired surgical margin around the nodule and maintaining a plane of dissection over the entire circumference of the tumor through the skin, subcutaneous tissues, and muscles until a surgical margin is achieved (90). The muscles, nerves, and blood vessels branching into the resection field are excised at the edge of the surgical margin. This technique is considered efficient in controlling recurrence, as STSs are often surrounded by a pseudocapsule, which can be invaded by tumor cells, which expand into the surrounding tissues. Thus, enlarging the surgical margin prevents microscopic disease from remaining *in situ*, preventing tumor recurrence (75).

**Figure 2:**
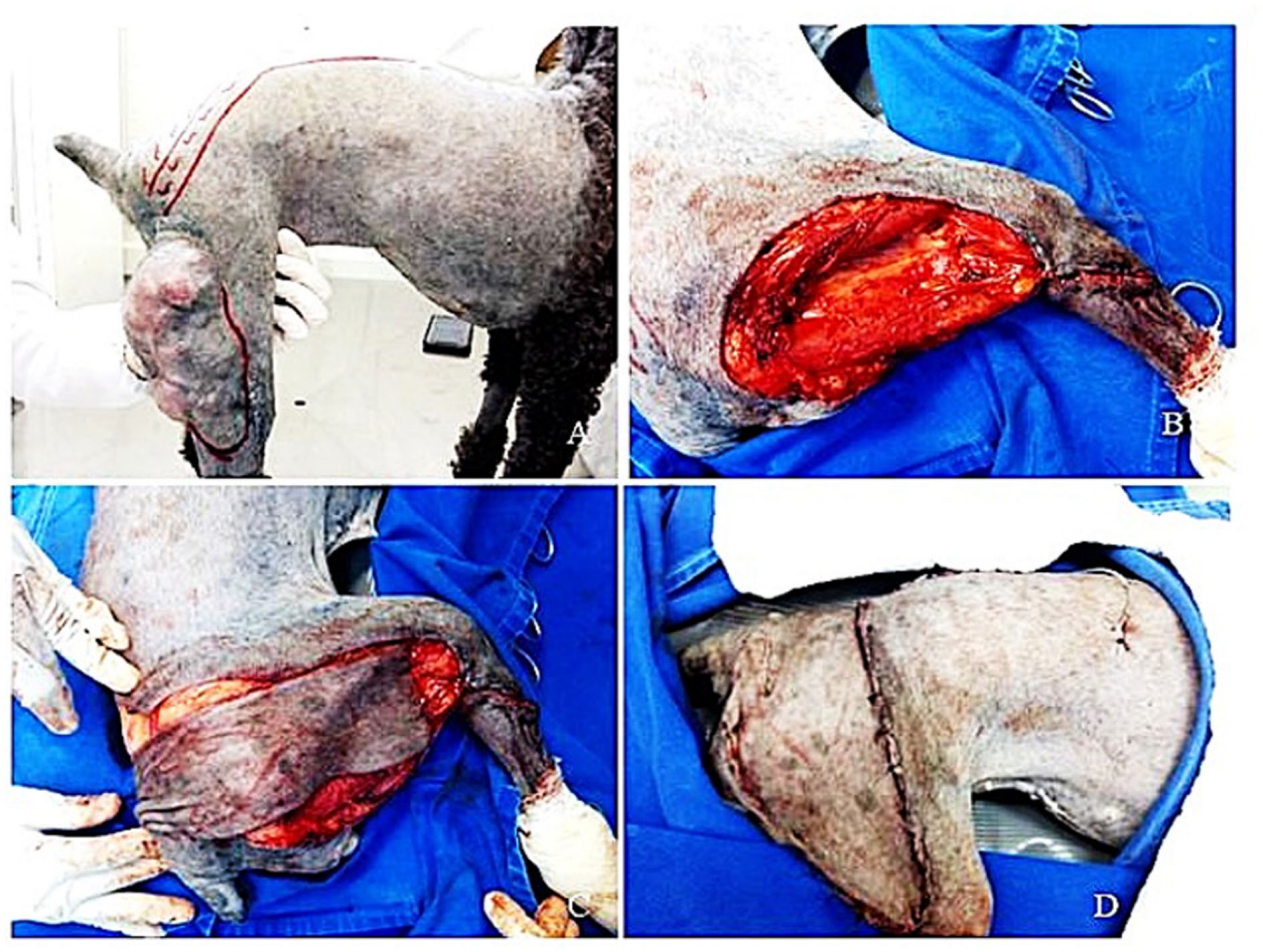
Surgical approach of a canine soft tissue tumor (STT) with fibrosarcoma as final diagnosis. **(A)** Macroscopic appearance of the STT in the pelvic forelimb in the region of quadriceps femoris muscle. **(B)** Oncological surgery with en bloc removal of the neoplastic lesion. **(C)** Creation of an axial pattern flap to reconstruct the surgical defect. **(D)** Final appearance of the sutures after complete tumor removal.

Due to the individual tumor biology of STSs, it is difficult to predict the exact extent of involvement of adjacent structures by the tumor; therefore, prescribing wide surgical margins in this type of approach has become standard for the treatment of this disease, avoiding the risk of recurrence (90). Given this information, there is a consensus that a wide surgical approach is recommended, following the principles of oncological surgery, and that the en bloc resection technique is effective and can be used in STS cases (Figure 2).

### Compartment resection

Occasionally, an STS arises within an individual muscle or muscle group, often presenting as a well-demarcated lesion surrounded by a pseudocapsule. In such cases, en bloc resection of the entire tissue compartment (e.g., a single muscle or group of muscles surrounding the tumor, excised from origin to insertion) may improve local tumor control and contribute to better postoperative pain management (8, 75). Complete removal of the compartment ensures that the tumor is surrounded by robust anatomical barriers on all sides, with the reactive zone and any satellite nodules contained within an entire natural compartment rather than in an arbitrarily measured block of tissue (75).

*Consensus recommendation*: the consensus group recommends compartment resection for soft tissue sarcomas confined to a single muscle or a well-defined muscle group, when anatomically feasible, to achieve complete excision and optimal local tumor control.

### Radiation therapy

Radiation therapy (RT) plays a key role in the multimodal management of canine soft-tissue mesenchymal tumors, particularly when complete surgical excision is not possible or when histopathology reveals incomplete margins. In veterinary oncology, RT is most commonly delivered with megavoltage cobalt-60 units or linear accelerators, using either conventional fractionation or hypofractionated schedules depending on treatment intent and availability (93). Treatment planning should always consider tumor location, volume, grade, and proximity to critical organs (94). Accurate immobilization, image-based planning, and appropriate dosimetry are essential to ensure a homogeneous dose distribution while minimizing exposure to normal tissues (95, 96). Acute side effects (erythema, desquamation, alopecia) typically arise during the second to third week of treatment and are self-limiting. Late effects such as fibrosis are uncommon when total doses ≤57 Gy with standard fractionation are used (93).

Adjuvant RT is recommended for patients with incompletely excised or narrow-margin STSs. The goal is eradication of microscopic residual disease and improved local control following surgery (97, 98). Treatment typically begins 2–4 weeks post-operatively, after adequate wound healing. Conventional fractionation schemes deliver a total dose of 45–57 Gy in 10–20 fractions, administered three to five times per week (99, 100).

Adjuvant RT improves local control in up to 70–80% of cases, with median survival times of 18–24 months and 1- and 3-year local control rates of 81 and 73%, respectively. Skin flaps or grafts should be carefully evaluated before planning, as altered vascularization can modify radiation tolerance and healing (97).

Definitive-intent RT is indicated for non-resectable, unresected, or recurrent STSs in which surgery is contraindicated or declined by the owner. The objective is durable local tumor control and prolonged survival. Definitive protocols are conventionally fractionated, delivering 48–54 Gy in 10–20 fractions. Modern image-guided or conformal techniques may be used where available (101), although stereotactic body RT (SBRT) remains limited in Brazilian veterinary settings and is not yet widely available.

In studies of dogs with STSs and gross disease treated with definitive RT (48–57 Gy delivered in 3 Gy fractions), approximately 40–60% achieved local control at 18–24 months (97, 101).

Neoadjuvant (preoperative) RT has recently emerged as a potential strategy to improve local control and facilitate surgical resection of canine soft-tissue sarcomas, paralleling established approaches in human oncology. However, veterinary data remain limited, with only one prospective study published to date. In that study, a preoperative hypofractionated RT protocol was evaluated in 12 dogs with STSs: 5 fractions of 6 Gy (total 30 Gy) were administered before surgery, which occurred 2–3 weeks after RT completion. Treatment was well tolerated, with moderate wound-healing complications (Grade II–III) in 25% of dogs and no severe late toxicities. Complete histologic excision (tumor-free margins) was achieved in 83% of cases, and a modest median tumor-volume reduction of 18% was observed prior to surgery. Short-term follow-up (median <18 months) showed 100% local control within the study period (101).

Palliative RT is appropriate for advanced, non-resectable, or metastatic STSs, and for patients with comorbidities or logistical constraints that preclude definitive therapy. The goals are pain relief, tumor stabilization, and improved quality of life. Hypofractionated regimens commonly used include 4 × 8 Gy once weekly, or 5–6 × 6 Gy twice weekly. These schedules reduce treatment time and anesthesia requirements while maintaining palliative efficacy. Clinical improvement is observed in approximately 50–80% of patients, with a median duration of response of 4–11 months (98).

A pilot study using SBRT for canine soft-tissue sarcomas, employing either 3 × 8 Gy or 5 × 6 Gy protocols, reported 1-year local control of 70%, with mild to moderate side effects. The authors concluded that hypofractionated, image-guided RT is a promising approach for non-resectable disease (102).

*Consensus recommendations*: this consensus recommends the use of radiation therapy as an adjuvant treatment for canine soft tissue mesenchymal tumors with incomplete or narrow surgical margins to eradicate microscopic residual disease and improve local tumor control. Definitive-intent RT is indicated for non-resectable, unresected, or recurrent tumors, while neoadjuvant RT may be considered in selected cases to facilitate surgical excision. Palliative RT should be reserved for advanced or metastatic tumors to relieve pain and improve quality of life. Treatment planning must be image-guided, ensuring precise dosimetry and protection of adjacent normal tissues.

### Lymphadenectomy

Lymphadenectomy for STSs is controversial as few studies have evaluated the importance of performing this procedure for these neoplasms. However, these neoplasms normally have a low probability of metastasis. Metastasis is more correlated with Grade III tumors, which have an approximately 40% chance of metastasizing (66, 90, 103). Furthermore, STSs normally present metastatic dissemination through the hematogenous route, and dissemination of neoplastic cells through the lymphatic organs is unlikely (90). Therefore, some authors recommend incisional biopsy and cytology before the surgical removal of sentinel lymph nodes in cases of Grade III STSs (21).

In a recent study with a large population of STS cases, lymph node metastasis occurred in 1.9% (2/105). In both cases, the patients had Grade III STSs and lymph node removal occurred due to lymph node enlargement. The authors followed the remaining 103 animals until death or at least 2 years postoperatively. None of the patients who did not undergo lymphadenectomy presented late lymph node enlargement during assessment (66).

*Consensus recommendation*: based on the current literature, this consensus recommends lymph node removal only if they are enlarged. We consider the metastatic rate of STSs to be low, and the lymph nodes appear to be enlarged when affected (66).

### Electrochemotherapy

**Figure 3:**
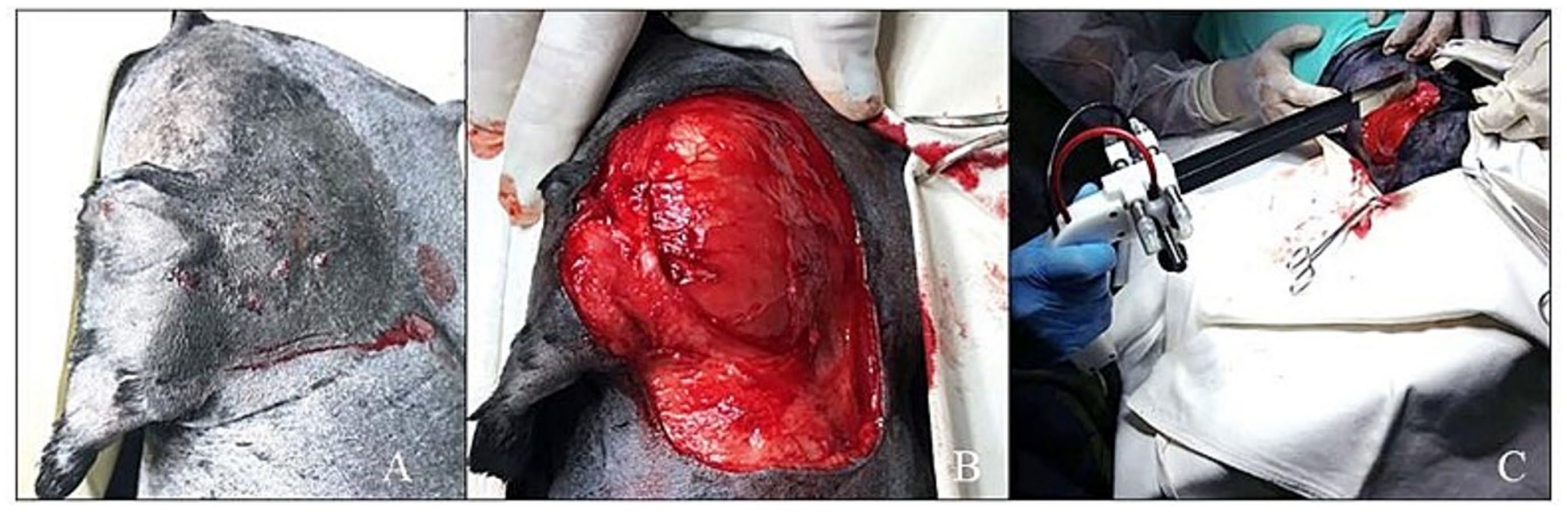
**(A)** Lesion in the cervical region of a dog diagnosed as Soft Tissue Sarcoma. **(B)** Surgical field after wide resection of the lesion. **(C)** Carrying out an electrochemotherapy procedure in the surgical field.

During our literature review, electrochemotherapy (ECT) was identified as complementary to surgical procedures (Figure 3) when radiation therapy is not available (63, 91, 104). As a primary antitumor therapy, insufficient evidence was available to indicate ECT for treating dogs with STSs. In a previous study, STTs in dogs were treated with intratumoral bleomycin and cisplatin; this had an objective response rate of 95%. In the same study, tumors smaller than 3 cm had a better response, and the association with ECT in tumors with a poor response to surgery or chemotherapy was considered positive (105). However, it is a relatively new technique, and more studies are necessary to reach any conclusion.

*Consensus recommendation*: based on the literature analysis, this consensus does not recommend the use of ECT as a sole treatment in dogs with STSs. ECT can be considered promising in association with surgery when radiation therapy is not possible. However, there is currently insufficient high-quality evidence (i.e., prospective controlled studies) demonstrating that ECT effectively eliminates tumor cells at surgical margins. Furthermore, surgery associated with ECT in deep margins does not replace radiotherapy.

### Chemotherapy

Chemotherapy has not shown significant benefit as a sole therapy for macroscopic STS. However, its use in both neoadjuvant and adjuvant settings has been explored, with potential roles in selected cases (106). The role of chemotherapy in STSs remains unclear, and most human and veterinary studies have failed to demonstrate a consistent survival advantage. This may be attributed to the heterogeneous nature of the disease, in which potential benefits for certain tumor subtypes may be masked by poor responses in others (62).

Chemotherapy is primarily reserved for Grade III tumors or cases with documented metastatic disease, as the overall metastatic rate for STSs is considered low. Metastasis is rarely reported in Grade I tumors, with rates ranging from 0 to 13% in Grade II, and 22 to 44% in Grade III tumors (107). In humans, the most used drugs for STS treatment are doxorubicin and ifosfamide, but response rates are <30%. In human medicine, chemotherapy as a single or combined agent increases disease-free time but does not change patient survival when compared to surgery as a single modality (62).

Doxorubicin is the most effective cytotoxic agent for use (1, 2). Protocols based on doxorubicin as a single drug, or in combination with cyclophosphamide, are the most used in dogs, with a response rate of 23% (106). “Continuous low-dose” (metronomic) chemotherapy is considered to slow progression by modulating the immunotolerance to the tumor and interrupting the development of the vascular supply that is supporting the tumor. Metronomic chemotherapy has been proposed to offer clinical benefits as quickly as conventional chemotherapy; however, clinical data are limited (108).

The combination of cyclophosphamide and piroxicam was evaluated in a metronomic manner in incompletely excised STSs. The 85 dogs studied were divided into two groups: 30 animals received cyclophosphamide (10 mg/m2) and piroxicam (0.3 mg/kg) daily, and 55 dogs did not receive any form of postoperative chemotherapy. Disease-free time was significantly longer in animals that received metronomic chemotherapy, suggesting that this modality may be a viable option with good local control results (109).

To evaluate the percentage and number of Treg cells and vascular microdensity in patients with STSs undergoing cyclophosphamide treatment with two metronomic regimens, dogs with STSs were enrolled in 2-dose cohorts and received cyclophosphamide at 12.5 or 15 mg/ m^2^ PO once daily for 28 days (108). Whole blood and tumor biopsy samples were obtained on days 0, 14, and 28 to assess changes in T lymphocyte subsets, using flow cytometry and tumor microvessel density (MVD). Cyclophosphamide at 12.5 mg/m^2^ significantly decreased Treg numbers from days 0 to 28, but there was no change in Treg percentage or tumor MVD. In dogs that received the 15.0 mg/m^2^ dose, both the number and percentage of regulatory Tcells (Tregs), as well as the MVD of the tumor, decreased significantly over the 28 days of the study (108).

No adverse events were observed in any dog during the study period. Up to 23% of dogs developed Grade 1 or 2 gastrointestinal toxicity, and 10–22% of dogs developed experimental sterile hemorrhagic cystitis. However, as a nonsteroidal anti-inflammatory drug (NSAID) was used, it is possible that the gastrointestinal signs were secondary to the NSAID and not to cyclophosphamide (108–110).

*Consensus recommendation*: this consensus recommends that chemotherapy should not be routinely employed as a primary treatment for canine STSs, given the lack of consistent evidence demonstrating a survival benefit. Its use may be considered in selected cases, particularly for Grade III tumors or those with documented metastasis, where systemic control is warranted. Doxorubicin-based protocols, alone or combined with cyclophosphamide, remain the preferred cytotoxic regimens, although reported response rates are modest (~20–30%). For incompletely excised tumors, metronomic chemotherapy using cyclophosphamide in combination with a nonsteroidal anti-inflammatory drug (e.g., piroxicam) may be considered to delay recurrence and modulate the tumor microenvironment, particularly through immunoregulatory and antiangiogenic mechanisms. However, clinical evidence remains limited, and treatment decisions should balance Enhanced potential benefits against toxicity risks. Future studies should focus on prospective, controlled trials to better define chemotherapeutic indications and identify molecular subgroups that may benefit most from systemic therapy.

### Immunotherapy

Cancer immunotherapy has changed the treatment landscape in human oncology, modified therapeutic algorithms for multiple malignancies, and become the primary treatment for metastatic disease. However, a significant proportion of patients are resistant to immunotherapy, and STSs have a limited response to immunotherapy (111).

Although the molecular characteristics of STSs are being investigated, our understanding of the events that occur in the tumor-immune system interaction in STSs is far from satisfactory. The phenotypic profile of immune infiltrates in these tumors should guide decision-making on the application of immunotherapy (112, 113). Furthermore, deciphering the signatures of immune cells within the tumor can discriminate responders from non-responders to immunotherapy, and different immunotherapeutic approaches can be combined based on the predominant cell subpopulations that infiltrate the STS (112, 113).

Regarding the diverse populations of immune cells that infiltrate the tumor microenvironment of STSs in humans, and therapeutic approaches targeting these cell populations (T cells, CD8 T cells [cytotoxic], CD4 T cells [helper], natural killer, macrophages, Tregs and FOXP3 T cells, the treatment of metastatic STS has not changed for decades, and with current conventional therapies, only a small chance of improving OS rates can be guaranteed (114). Infiltration of the microenvironment with CD8 + T cells and expression of immune checkpoint molecules have become prerequisites for effective immunotherapy with immune checkpoint inhibitors (115).

Pleomorphic sarcoma and myxofibrosarcoma have some of the highest levels of CD8 + T cell infiltration and the highest expression of PD-1 (114). Therefore, these tumors are expected to benefit equally from immune checkpoint inhibitors. However, this has not been studied in the field of veterinary medicine. Immunotherapies can be easily combined, and chemotherapy can serve as a powerful tool to sensitize the tumor microenvironment to immunotherapy in chemo-resistant tumors (116). The combination of chemotherapy and immunotherapy should be a target of future research within the scope of comparative oncology.

In veterinary medicine, there is little data on the use of immunotherapy in dogs with STSs. A xenogeneic (human recombinant) anti-vascular endothelial growth factor (VEGF) vaccine was evaluated in nine dogs with STSs, regarding safety and efficacy on angiogenesis and tumor growth. However, the response rate was only 30% (three animals experienced partial remission, whereas six had progressive disease). Among these animals, three showed a reduction in plasma VEGF concentration, prompting the authors to suggest that this form of immunotherapy could serve as an adjunct alternative in the treatment of STSs (117).

*Consensus recommendation*: this consensus recognizes that the role of immunotherapy in the treatment of canine STSs remains largely unexplored, and its clinical efficacy is yet to be demonstrated. Given the low response rates reported in both human and veterinary studies, immunotherapy should currently be considered an experimental or adjunctive option, preferably within controlled research settings.

### Other therapies

Liposomal clodronate (LC), a first-generation bisphosphonate encapsulated in liposomes, is used to reduce the number of macrophages in animals with cancer. There is some evidence in mice that systemic administration of LC efficiently depletes macrophages at a variety of body sites (118, 119). The introduction of clodronate into the macrophage cytoplasm appeared to induce rapid onset apoptosis through competition for ATP binding to cellular substrates. The antitumor activity of LC has been attributed primarily to the depletion of tumor associated macrophages, and secondarily to the inhibition of tumor angiogenesis (120).

Intravenous 2.5 mg/kg liposomal clodronate, in a volume of 0.5 mL/kg over 60 min via an indwelling venous catheter, was administered to dogs once a week (*N =* 11) or once every 2 weeks (*N =* 2) for a total of six or four treatments, respectively. In five dogs with STSs, a series of LC infusions was able to deplete tumor- associated macrophages (TAMs) and suppress circulating IL-8 concentrations. Although LC treatment has not been associated with clinically significant tumor growth responses, these studies have demonstrated the potential of macrophage-depleting therapies in animals with spontaneous cancer, including dogs and humans (121).

### Paraneoplastic syndromes

Paraneoplastic syndromes (PNS) are clinical disorders associated with malignant neoplasms that are not directly attributable to the physical presence of the tumor or its metastases (122). These syndromes result from the production of biologically active substances such as hormones, peptides, or cytokines by neoplastic cells, or from immune-mediated mechanisms triggered by the tumor (122, 123).

Paraneoplastic hemolytic anemia may also occur via immune-mediated or microangiopathic mechanisms. It is normally associated with tumors with significant neovascularization; when blood passes through these tumors, the red blood cells collected by the immune system are damaged. In this case, the anemia is characterized as regenerative with the presence of spherocytes, and treatment includes surgical removal of the neoplasm; however, in some cases where a surgical procedure is not possible, immunosuppressive drugs and transfusion can be used as emergency measures (124). Anemia, when associated with chronic diseases, is caused by a decrease in erythrocyte lifespan and iron availability, as well as a decrease in erythropoiesis. It is classified as normocytic, normochromic, non-regenerative, and is generally mild. Resection of the neoplasm associated with the PNS is the best option for resolving clinical conditions (122).

Research on STSs has described an associated tumor-induced osteomalacia in dogs. The associated mechanism is that some mesenchymal neoplasms can modify the physiological metabolism of phosphorus in the body through the production of molecules such as fibroblast growth factor 23, which lead to renal loss of phosphate ions and serum absorption or availability of 1,25(OH)_2_ vitamin D3, which causes PNS. The authors of this study evaluated the expression of fibroblast growth factor 23 in 49 cases of STSs and in the normal tissues of dogs. They detected the expression of growth factor 23 in the bone, lungs, kidneys, lymph nodes, and thymus. Fifteen of 49 tumors (31%) expressed fibroblast growth factor 23, three of which had high relative expression and some features that resembled human mesenchymal tumors expressing phosphatonin (125).

Other PNS conditions, such as leukocytosis due to acute myeloid hyperplasia in the bone marrow in a case of liposarcoma without ongoing infection, have also been described in isolated case reports of STTs in dogs (126).

In humans, a severe leukemoid reaction has already been associated with an STT in a 62-year-old woman. After surgical removal of the primary mass, the reaction decreased immediately after the procedure (127).

## Prognosis

The prognosis for most dogs with STS is favorable when complete resection is achieved. However, recurrence has been reported in 7 to 75% of cases, depending on tumor grade and surgical margins (4). Metastatic rates for canine soft tissue sarcomas vary according to histologic grade. Low-grade (Grade I) tumors have a metastatic rate of <10%, whereas intermediate-grade (Grade II) tumors show rates ranging from 10 to 20% (66). High-grade (Grade III) tumors have the highest risk, with metastatic rates reported between 40 and 50% (21). Overall, approximately 20 to 30% of dogs treated for STS will eventually die due to disease progression. Continued efforts to improve therapeutic strategies and to identify dogs with the highest risk of metastasis remain crucial (63).

Two prognostic factors were identified in a study of 236 animals. Tumor free surgical margins are a predictive factor for disease-free period and the most important factor for tumor recurrence, together with the histological grade (82). Grade I tumors have low recurrence rates after surgical excision; however, a recurrence rate of 7% is reported when the margins are small, even for tumors of this classification. Metastasis is rare, with the most common sites being the lung fields and regional lymph nodes (82, 88). When completely excised, Grade II tumors have relatively low recurrence rates; however, they recur more often than Grade I tumors, particularly when surgical margins are narrow. Additionally, the disease-free interval for Grade II tumors is generally shorter than that observed for Grade I tumors (82, 128).

Grade III tumors are the least frequent, corresponding to 7 to 17% of all STSs that affect the dermis of dogs, and have a high potential for recurrence and metastasis (82, 128, 129). In cases where the margins of excision were wide and free of tumor cells, the percentage of local recurrence was 7 up to 17% (82, 130, 131). Grade III STSs with small margins have a higher recurrence rate (40% up to 75%) compared to that of Grade I and II tumors, and complete excision of Grade III tumors is more complex than that of tumors of other grades (82).

In addition to grade, the MI is related to the prognosis of animals with STSs (66). The MI provides relevant information regardless of the grade, and the STS is divided into three scores: Score 1, when there are 0 to 9 mitotic figures in 10 fields of highest magnification (400 x); Score 2, when there are 10 to 19 mitotic figures in 10 fields of highest magnification (400 x); and Score 3, when there is a number equal to or greater than 20 mitotic figures in 10 fields of highest magnification (400 x) (132). High MI is associated with recurrence, metastasis, and decreased survival. Survival time is 150 to 343 days for MI ≥ 9, whereas for MI < 9, the average survival time is 860 and 1,138 days (132).

In another evaluation of average survival time according to MI for STSs, survival of 236 days was observed for an MI > 20, 532 days for an MI between 10 and 19, and 1,444 days for an MI < 10. Metastasis is five times more frequent in tumors with an MI equal to or greater than 20 mitotic figures in 10 high-power fields (400X). The death rate was 2.6 times higher in patients with MIs > 20 than in those with MIs < 20 (107).

In a recent study, animals with STSs and MIs greater than or <5 showed a significant difference in survival. Dogs with a mitotic count >5 had a mean overall survival of 256 days, whereas those with a mitotic count ≤5 exhibited a longer mean overall survival of 402 days. Furthermore, the survival time was shorter in patients with Grade III tumors than in those with Grade I or II tumors. The average OS was 445 days for patients with Stage I, 375 for Stage II, 224 for Stage III, and 278 for Stage IV. Patients with Stage III and IV tumors had shorter survival times than those with Stage I and II tumors. Patients with lymph node metastases survived not more than 120 days, whereas those without metastases survived more than 363 days (66).

In addition to clinical and histological parameters, immunohistochemical markers are frequently used as prognostic factors in human and veterinary medicine. Higher expression of cell proliferation markers, such as AgNOR and Ki-67, are also prognostic factors for decreased survival times (129). These techniques provide additional information regarding the proliferation status during tumor evaluation, and are routinely used to obtain prognostic information for several human neoplasms. However, it is unclear whether these and other markers provide adequate prognostic information in dogs (129).

Tumor size is also considered a prognostic factor. Larger tumors are more difficult to remove than smaller ones and tumor size directly affects the surgical procedure and the possibility of residual disease (66). Several canine and human studies have suggested that tumors >5 cm are associated with shorter disease-free survival times than tumors smaller than this size (7, 103). In a study, the average tumor size was 6.4 cm, which directly correlated with survival time that was greater in dogs with T < 6.4 cm (66).

In general, tumor grade, assessment of surgical margins, lymph node metastasis, and tumor size are the main prognostic indicators for average survival time.

*Consensus recommendation*: We recommend applying the established histopathological grading system (4), dividing tumors into Grades I, II, and III, and considering a mitotic count threshold of 5 (51) as key prognostic factors for canine STSs. Currently, there is no evidence supporting the use of immunohistochemistry as a prognostic tool in this tumor type.

## Conclusion

STSs are considered a heterogeneous group of tumors, with a need for standardization of different aspects, such as assessment of necrosis and its impact on tumor grading, molecular characterization of the different tumor subtypes, and new prognostic factors to indicate more aggressive tumors.

## Glossary

ABROVET: Brazilian Association of Veterinary Oncology
CT: Computed tomography
M: Distant metastasis
ECT: Electrochemotherapy
ERs: Estrogen receptors
IHC: Immunohistochemistry
LC: Liposomal clodronate
MVD: Microvessel density
MC: Mitotic count
NSAID: Nonsteroidal anti-inflammatory drug
OS: Overall survival
PNS: Paraneoplastic syndromes
PNST: Peripheral nerve sheath tumors
PWTs: Perivascular wall tumors
PDGF-A: Platelet-derived growth factor alpha
PDGFR- **β**: Platelet-derived growth factor β
PRs: Progesterone receptors
RT: Radiation therapy
N: Regional nodal involvement
Tregs: Regulatory Tcells
SMA: Smooth muscle actin
STN: Soft tissue neoplasm
STTs: Soft tissue sarcomas
STTs: Soft Tissue Tumors
SBRT: Stereotactic body RT
TAMs: Tumor- associated macrophages
T: Tumor size
UPSs: Undifferentiated pleomorphic sarcomas
UPSs: Undifferentiated pleomorphic sarcomas
VEGF: Vascular endothelial growth factor
WHO: World Health Organization
