# Supplementary material for: Canine cutaneous and subcutaneous soft tissue sarcoma in dogs: a consensus report from the Brazilian association of veterinary oncology
Source: Front Vet Sci. 2026 May 7;13:1750148. doi: 10.3389/fvets.2026.1750148 (PMC13192336; doi:10.3389/fvets.2026.1750148)
Supplement: Supplementary file 1 [file Table_1.DOCX]

**Supplementary Table S1**. Common molecular and immunohistochemical markers reported in canine soft tissue sarcomas.

| **Marker / Gene** | **Type of marker** | **Main biological function** | **Chromosomal location (Canis lupus familiaris)** | **Relevance in soft tissue tumors** |
| --- | --- | --- | --- | --- |
| **Ki67 (MKI67)** | Proliferation marker | Nuclear protein expressed during all active phases of the cell cycle; indicator of cellular proliferation | CFA27 | High Ki67 index correlates with higher tumor grade and aggressive biological behavior |
| **Endosialin (CD248)** | Tumor stromal marker | Transmembrane glycoprotein expressed in activated fibroblasts and tumor-associated stromal cells | CFA9 | Associated with tumor proliferation, stromal remodeling, and metastatic potential |
| **FoxP3** | Transcription factor | Master regulator of regulatory T lymphocyte differentiation and immune tolerance | CFA7 | Its expression in tumors may be associated with immune evasion mechanisms |
| **COX-2 (PTGS2)** | Inflammatory enzyme | Catalyzes prostaglandin synthesis and mediates inflammatory responses | CFA7 | Frequently overexpressed in tumors; associated with inflammation, angiogenesis, and tumor progression |
| **PDGF-A (PDGFA)** | Growth factor | Stimulates proliferation and migration of fibroblasts and mesenchymal cells | CFA6 | Promotes tumor growth and stromal activation |
| **PDGFR-α (PDGFRA)** | Receptor tyrosine kinase | Mediates signaling from PDGF ligands, activating proliferation and survival pathways | CFA13 | Activation contributes to tumor progression and may represent a therapeutic target |
| **Integrin αV (ITGAV)** | Cell adhesion receptor | Mediates cell-extracellular matrix interaction and regulates migration and invasion | CFA9 | Associated with tumor invasion, angiogenesis, and metastatic capacity |
| **HRAS** | Oncogene | Small GTPase involved in MAPK signaling and cell proliferation | CFA18 | Activating mutations drive tumorigenesis in multiple cancers |
| **KRAS** | Oncogene | Regulates cell proliferation and survival through MAPK and PI3K pathways | CFA13 | Mutations are common drivers in several cancers but are rarely reported in canine fibrosarcoma |
| **NRAS** | Oncogene | Member of the RAS family regulating cellular growth and differentiation | CFA5 | Oncogenic mutations promote uncontrolled cell proliferation |
